# Increased Expression of TGF-β Signaling Components in a Mouse Model of Fibrosis Induced by Submandibular Gland Duct Ligation

**DOI:** 10.1371/journal.pone.0123641

**Published:** 2015-05-08

**Authors:** Lucas T. Woods, Jean M. Camden, Farid G. El-Sayed, Mahmoud G. Khalafalla, Michael J. Petris, Laurie Erb, Gary A. Weisman

**Affiliations:** 1 Department of Biochemistry, University of Missouri, Columbia, Missouri, United States of America; 2 Christopher S. Bond Life Sciences Center, University of Missouri, Columbia, Missouri, United States of America; 3 Department of Nutritional Sciences and Exercise Physiology, University of Missouri, Columbia, Missouri, United States of America; Cincinnati Children's Hospital Medical Center, UNITED STATES

## Abstract

Transforming growth factor-β (TGF-β) is a multi-functional cytokine with a well-described role in the regulation of tissue fibrosis and regeneration in the liver, kidney and lung. Submandibular gland (SMG) duct ligation and subsequent deligation in rodents is a classical model for studying salivary gland damage and regeneration. While previous studies suggest that TGF-β may contribute to salivary gland fibrosis, the expression of TGF-β signaling components has not been investigated in relation to mouse SMG duct ligation-induced fibrosis and regeneration following ductal deligation. Following a 7 day SMG duct ligation, TGF-β1 and TGF-β3 were significantly upregulated in the SMG, as were TGF-β receptor 1 and downstream Smad family transcription factors in salivary acinar cells, but not in ductal cells. In acinar cells, duct ligation also led to upregulation of snail, a Smad-activated E-cadherin repressor and regulator of epithelial-mesenchymal transition, whereas in ductal cells upregulation of E-cadherin was observed while snail expression was unchanged. Upregulation of these TGF-β signaling components correlated with upregulation of fibrosis markers collagen 1 and fibronectin, responses that were inhibited by administration of the TGF-β receptor 1 inhibitors SB431542 or GW788388. After SMG regeneration following a 28 day duct deligation, TGF-β signaling components and epithelial-mesenchymal transition markers returned to levels similar to non-ligated controls. The results from this study indicate that increased TGF-β signaling contributes to duct ligation-induced changes in salivary epithelium that correlate with glandular fibrosis. Furthermore, the reversibility of enhanced TGF-β signaling in acinar cells of duct-ligated mouse SMG after deligation indicates that this is an ideal model for studying TGF-β signaling mechanisms in salivary epithelium as well as mechanisms of fibrosis initiation and their resolution.

## Introduction

The salivary glands are exocrine glands that secrete saliva into the oral cavity where components of saliva aid in digestion and prevent oral infection [1]. In humans, the majority of saliva is secreted from the parotid, submandibular and sublingual glands with minor contributions from numerous, small accessory glands. For saliva production, activation of muscarinic receptors on the basolateral membrane of acinar cells results in fluid secretion into the ductal lumen where the ion content is modulated as saliva travels along a series of collecting ducts into the main secretory duct which empties into the oral cavity [1]. Salivary dysfunction can significantly decrease quality of life and leads to dry mouth, oral infection and poor nutrition [2]. Two primary causes of salivary dysfunction in humans are Sjögren’s syndrome (SS), an autoimmune disease characterized by lymphocytic infiltration of the salivary gland and production of autoantibodies, and γ-radiation-induced dysfunction, an unintended consequence of treatment for head and neck cancers [3, 4]. Current treatments for salivary hypofunction (*i*.*e*., xerostomia) include administration of sialogogues and saliva substitutes; however, these approaches are limited to treating the symptom and not the cause of xerostomia [5]. New strategies to treat xerostomia are being investigated to regenerate salivary glands and restore normal levels of saliva secretion [6, 7]. Understanding the underlying mechanisms of salivary gland inflammation in SS and radiation therapy that result in tissue damage could reveal novel targets to prevent salivary gland degeneration and promote restoration of functional tissue.

Salivary gland duct ligation and subsequent deligation is a well-studied animal model of salivary gland inflammation (ligation) and regeneration (deligation) [8–14]. In this model, the main excretory duct of the submandibular gland (SMG) is surgically occluded and after a set period of time (~1–30 days) the process is reversed. Within 24 hours of duct ligation, immune cells begin to infiltrate the gland and acinar cells begin to atrophy, lose expression of acinar proteins and eventually undergo apoptosis [8, 10, 11, 15–17]. Ductal cells, subsets of which have been proposed to be salivary progenitor cells that can regenerate the gland, remain intact and proliferative during ligation [11, 18, 19]. Additionally, ligation causes salivary glands to become fibrotic [20]. Interestingly, once the gland is deligated, it begins to regenerate and regains saliva production [12, 14, 21]. Studies in rats have demonstrated that SMGs regenerate histologically and functionally upon deligation, even after >30 days of duct ligation [21]. The ability of the SMG to recover from severe inflammatory and fibrotic events following ligation has made duct ligation a useful experimental approach for investigating mechanisms underlying salivary gland inflammation and regeneration.

Previous studies with liver, lung and kidney have elucidated a key role for transforming growth factor-β (TGF-β) in regulating fibrosis and tissue regeneration [22–25]. Three isoforms of TGF-β have been identified and denoted as TGF-β1, TGF-β2 and TGF-β3. These cytokines are unique in their mechanism of latent activation [26]. TGF-β is produced as a proprotein that is proteolytically cleaved into TGF-β and the latency associated peptide (LAP). Following cleavage, TGF-β and LAP remain covalently bound and LAP forms disulfide bonds with the latent TGF-β binding protein (LTBP). This Large Latent Complex (LLC), consisting of TGF-β, LAP and LTBP, is then secreted from the cell [26]. The release of active TGF-β from the LLC occurs through proteolytic cleavage by matrix metalloproteases, disruption of non-covalent interactions by thrombospondin-1 and interaction of LAP with integrins [27–29]. Once TGF-β is released, activation of the canonical TGF-β signaling pathway occurs through binding of TGF-β to TGF-β receptor 2 (TGF-β R2), which then dimerizes with TGF-β receptor 1 (TGF-β R1) leading to intracellular phosphorylation and activation of the transcription factors Smad2 and Smad3 [30]. Along with Smad4, these transcription factors induce cellular responses to extracellular TGF-β by activating several DNA-binding transcription factors including Snai1 (Snail) and Snai2 (Slug) [31]. Snail and Slug have been well-described as mediators of TGF-β-induced epithelial-mesenchymal transition (EMT) [32–34]. Non-canonical TGF-β signaling pathways also have been shown to contribute to TGF-β-induced EMT, specifically through the activation of TGF-β-activated kinase 1 (TAK1) and TAK-1-binding protein (TAB1) [35, 36].

EMT is a cellular mechanism by which epithelial cells dedifferentiate from their epithelial status to a mesenchymal-like phenotype [37]. This process has been suggested to play a major role in the metastasis of cancer cells, tissue fibrosis and regeneration [38–40]. During EMT, epithelial proteins such as E-cadherin and zona occludens-1 (ZO-1) are downregulated while mesenchymal proteins such as vimentin and α-smooth muscle actin (α-SMA) and fibrotic proteins such as fibronectin and collagen 1 are upregulated [41]. The cells undergoing EMT then become mesenchymal-like, increasing their capacity for migration and redifferentiation. Snail functions in EMT by repressing the expression of epithelial E-cadherin [32, 42, 43]. Given the importance of TGF-β signaling to EMT and fibrosis and considering previous reports on the role of TGF-β in liver and kidney regeneration, we explored whether expression of TGF-β and its downsteam signaling proteins is altered in the SMG ligation/deligation model of salivary gland inflammation and regeneration. Our results demonstrate increased expression of components of the TGF-β signaling pathway in salivary gland epithelium during SMG duct ligation and, interestingly, this response is more pronounced in acinar cells compared to ductal cells. Furthermore, we found increased expression of E-cadherin and fibrosis markers during SMG ligation that, along with proteins in the TGF-β signaling cascade, return to normal levels following deligation, which correlates with restoration of normal tissue architecture. We further demonstrate that treatment of mice with the TGF-β R1 inhibitors SB431542 or GW788388 significantly reduces the upregulation of the fibrosis markers collagen 1 and fibronectin caused by SMG duct ligation. Thus, this study demonstrates that the SMG ligation/deligation model is an excellent system for investigating reversible TGF-β-mediated signaling mechanisms in epithelium as well as mechanisms of fibrogenesis and fibrosis resolution.

## Materials and Methods

### Reagents

TRIzol Reagent, AlexaFluor 594 goat anti-rabbit IgG antibody, AlexaFluor 594 donkey anti-rabbit IgG antibody, AlexaFluor 488 donkey anti-goat IgG antibody and Hoechst 33258 nuclear stain were purchased from Life Technologies (Grand Island, NY). Rabbit anti-aquaporin-5 polyclonal antibody (178615) was purchased from EMD Millipore (Billerica, MA). Rat anti-CD45 monoclonal antibody (30-F11) was purchased from Biolegend (San Diego, CA). Rabbit anti-TGF-β1/2/3 polyclonal antibody (3771), rabbit anti-Smad2/3 monoclonal antibody (D7G7), rabbit anti-phospho-Smad2/3 monoclonal antibody (D27F4) and rabbit anti-E-cadherin monoclonal antibody (24E10) were purchased from Cell Signaling Technology (Danvers, MA). Rabbit anti-Snail polyclonal antibody (NBP1-19529) was purchased from Novus Biologicals (Littleton, CO). Rabbit anti-TGF-β R1 polyclonal antibody (H-100), goat anti-aquaporin-5 polyclonal antibody (G-19) and horseradish peroxidase-conjugated goat anti-rabbit IgG antibody were purchased from Santa Cruz Biotechnology (Santa Cruz, CA). The TGF-β R1 inhibitors SB431542 and GW788388 were purchased from Tocris Bioscience (Bristol, United Kingdom). All other reagents were purchased from Sigma-Aldrich (St. Louis, MO), unless stated otherwise.

### Animals and ethics

C57BL/6 mice were purchased from Jackson Laboratories (Bar Harbor, ME) and bred at the Christopher S. Bond Life Sciences Center Animal Facility of the University of Missouri, Columbia, MO. Animals were housed in vented cages with 12 h light/dark cycles and received food and water *ad libitum*. Age-matched 6–8 week old male mice were utilized for all experiments. All surgeries were performed following anesthesia by intraperitoneal injection with Avertin (0.75 mg/g mouse weight). Euthanasia was performed by terminal anesthesia followed by cervical dislocation and all efforts were made to minimize suffering. The protocol for this study was approved by the University of Missouri Animal Care and Use Committee (Protocol Number: 7880).

### Ligation and deligation of the SMG main excretory duct

Unilateral ligation of the SMG main excretory duct was performed as previously described [9]. Briefly, mice were anesthetized by intraperitoneal injection with Avertin (0.75 mg/g mouse weight) and the main excretory duct on one side of the neck was dissected and separated from surrounding connective tissue under a surgical stereoscope. The duct was ligated using surgical sutures with particular care taken to avoid ligation of surrounding blood vessels and nerves. The incision was closed using surgical clamps and the mice were allowed to recover. After 7 days, mice were either subjected to SMG deligation or anesthetized with isoflurane in a chamber and euthanized by cervical dislocation. For SMG deligation, mice were anesthetized (as above) and, following careful dissection of the neck, the surgical suture was removed from the SMG duct and the incision was closed using surgical clamps. Following 28 days of recovery, mice were anesthetized and euthanized (as above). Then, 7 day-ligated glands with or without a 28 day deligation and contralateral control glands were excised and processed for real-time PCR (RT-PCR), Western analysis or immunofluorescence as described below. For TGF-β R1 inhibitor studies, mice received intraperitoneal injection of SB431542 (20 mg/kg mouse weight in DMSO), GW788388 (2 mg/kg mouse weight in DMSO) or DMSO only as a vehicle control directly after SMG duct ligation and 4 days post-ligation. Seven day ligated and contralateral control glands with or without inhibitors were then collected and processed for RT-PCR analysis, as described below.

### Real-time PCR

Ligated, deligated and contralateral control glands were homogenized in TRIzol reagent. Following a 5 min incubation at room temperature, chloroform (0.2 ml/ml TRIzol) was added and samples were incubated for 5 min at room temperature. Samples were centrifuged at 12,000 x g for 15 min at 4°C and RNA isolation from the resulting aqueous phase was performed using the RNeasy Plus Mini Kit (Qiagen, Valencia, CA). cDNA was prepared from 1 μg of purified RNA using RNA to cDNA EcoDry Premix (Clontech Laboratories, Mountain View, CA). Specific Taqman primers for mouse *TGF-β1*, *TGF-β2*, *TGF-β3*, *TAK1 (MAP3K7)*, *TAB1*, *Snail* (*Snai1*), *Slug* (*Snai2*), *fibronectin* (*Fn1*), *collagen 1* (*Col1a1*), *E-cadherin* (*Cdh1*) and *18S* were purchased from Applied Biosystems (Foster City, CA) and used for RT-PCR on an Applied Biosystems 7500 Real-Time PCR machine. For data analysis, mRNA expression of target genes was normalized to 18S ribosomal RNA as an internal control.

### SDS-PAGE and western blot analysis

Ligated, deligated and contralateral control SMGs were homogenized in Tissue Protein Extraction Reagent (Thermo Scientific, Rockford, IL) containing protease inhibitor cocktail (Sigma-Aldrich). Samples were centrifuged at 10,000 x g for 5 min to pellet cellular debris, supernatants were collected and the protein concentration was measured using a Nanodrop 1000 spectrophotometer. Following protein concentration normalization, samples were combined 1:1 with 2X Laemmli Buffer (20 mM sodium phosphate, pH 7.0, 20% (v/v) glycerol, 4% (w/v) SDS, 0.01% (w/v) bromophenol blue and 100 mM DTT) and subjected to Western blot analysis, as previously described [8]. Briefly, samples containing 50 μg total protein were subjected to 7.5% (w/v) SDS-PAGE and transferred to nitrocellulose membranes. As a loading control, membranes were stained with Ponceau S solution (0.1% (w/v) Ponceau S in 5% (v/v) acetic acid) for 5 min followed by 2 washes in 5% (v/v) acetic acid. Membranes were then washed in Tris-buffered saline (pH 7.4) containing 0.1% (v/v) Tween-20 (TBST), blocked for 1 h with 5% (w/v) non-fat dry milk in TBST and incubated with rabbit anti-pro-TGF-β1/2/3 (diluted 1:1,000 in TBST), rabbit anti-Smad2/3 antibody (diluted 1:1,000 in TBST) or rabbit anti-phospho-Smad2/3 antibody (diluted 1:1,000 in TBST) for 16 h at 4°C. Membranes were then washed in TBST and incubated with horseradish peroxidase-conjugated goat anti-rabbit IgG antibody (1:2,000 dilution in TBST) at room temperature for 1 h. Protein bands were visualized using enhanced chemiluminescence reagent (Thermo Scientific) and detected on X-ray film.

### Immunofluorescence and brightfield microscopy

Immunofluorescence microscopy was performed as previously described [8]. Briefly, ligated, deligated and contralateral control SMGs were snap frozen in 2-methylbutane cooled with liquid nitrogen. Glands were then equilibrated to -20°C, cut into 8 µm sections using a Leica CM1900 cryostat and adhered to microscope slides. Sections were then fixed with 4% (v/v) paraformaldehyde in PBS, pH 7.4, for 20 min, washed three times in PBS and incubated in blocking buffer (5% (v/v) goat serum, 10 µM digitonin in PBS) for 2 h at room temperature. Sections were then incubated for 16 h at 4°C with rabbit primary antibodies specific for aquaporin-5, CD45, TGF-β R1, Smad2/3, phospho-Smad2/3, Snail or E-cadherin (diluted 1:250 in blocking buffer). Primary antibody specificity for aquaporin-5 [44], CD45 [45], TGF-β R1 [46], Smad2/3 and phospho-Smad2/3 [47], Snail [48] and E-cadherin [49] was determined, as cited, and sections incubated with only secondary antibody served as negative controls where no fluorescence was observed. Following three washes in PBS, sections were incubated with AlexaFluor 594 goat anti-rabbit IgG, AlexaFluor 488 goat anti-rabbit IgG or AlexaFluor 594 goat anti-rat IgG (diluted 1:1,000 in blocking buffer) for 1 h at room temperature. Sections were then washed three times in PBS and stained with the nuclear dye Hoechst 33258 (diluted 1:5,000 in PBS) for 5 min at room temperature. Following three washes in PBS, slides were mounted and dried. For dual-immunofluorescence analysis, goat serum in blocking buffer was replaced with donkey serum and, following a 2 h incubation in blocking buffer, SMG sections were treated with goat anti-aquaporin-5 antibody (diluted 1:250 in blocking buffer) and the indicated rabbit primary antibody for 16 h at 4°C. Then, AlexaFluor 594 donkey anti-rabbit IgG and AlexaFluor 488 donkey anti-goat IgG secondary antibodies (both diluted 1:1000 in blocking buffer) were added to washed sections for 1 h at room temperature. Fluorescence was visualized using a Nikon Ti-E inverted microscope equipped with appropriate filters.

For brightfield microscopy, ligated, deligated and contralateral control SMGs were placed in 4% (v/v) paraformaldehyde in PBS at 4°C for 24 h followed by 70% (v/v) ethanol for 24 h at 4°C. Samples were then sent to IDEXX RADIL (Columbia, MO) where glands were embedded in paraffin, cut into 5 μm sections and subjected to hematoxylin and eosin or Masson’s trichrome staining. Hematoxylin and eosin-stained slides were visualized on an AMG EVOS XL Core brightfield microscope and Masson’s trichrome-stained slides were visualized on an Olympus Vanox AHBT3 brightfield microscope at the University of Missouri Molecular Cytology Core Facility. Low magnification images of whole SMG sections were generated from multiple 4X magnification images stitched together with Leica LAS3.1 imaging software.

### Statistical analysis

Quantitative results are presented as means ± S.E.M. of data from three or more independent experiments. Statistical significance was defined as *P*<0.05 as calculated by one-way ANOVA with repeated measures post-test where appropriate or by a two tailed t-test, using GraphPad Prism software.

## Results

### Regeneration of duct-ligated SMG following deligation

**Fig 1:**
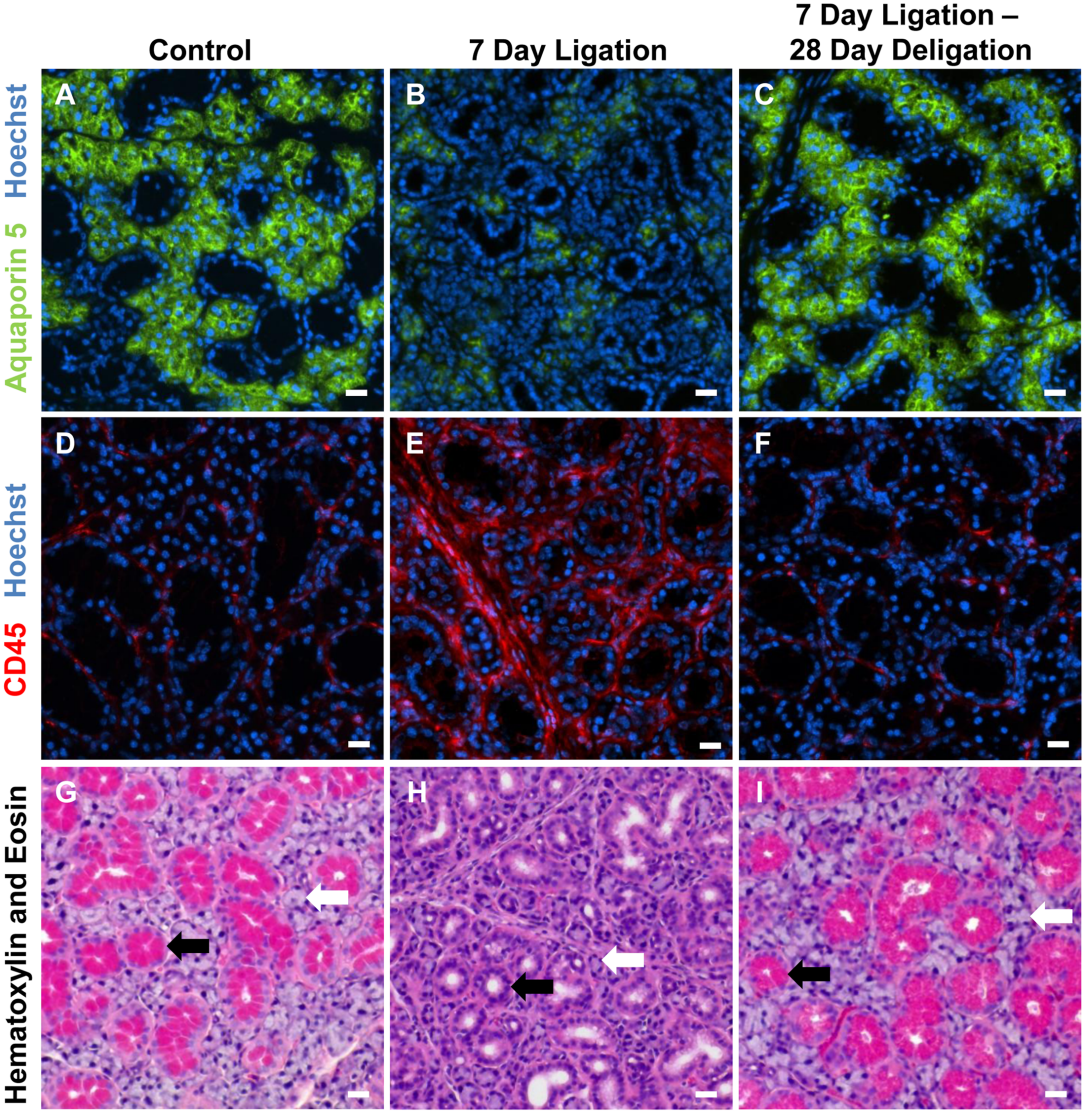
Submandibular gland ductal ligation followed by deligation results in reversible acinar cell atrophy, immune cell infiltration and glandular fibrosis. (A, D, G) Control SMG, (B, E, H) 7 day duct-ligated SMG and (C, F, I) 7 day duct-ligated SMG followed by deligation and recovery for 28 days were subjected to immunofluorescence staining with (A-C) antibodies to the acinar cell marker AQP5 (green) and Hoechst nuclear stain (blue), (D-F) antibodies to the pan-immune cell marker CD45 (red) and Hoechst nuclear stain (blue) and (G-I) hematoxylin and eosin. Results indicate that ligation of the main SMG excretory duct induces (B) loss of acinar cells, (E) substantial immune cell infiltration and (H) atrophy of acinar cells (white arrow) and loss of secretory granules (pink) within ductal cells (black arrow) that is reversed by subsequent deligation (C, F, I). Images are representative of results from at least 3 independent experiments and scale bar = 20 μm.

The SMG excretory duct ligation/deligation model has long been used for the study of salivary gland inflammation and regeneration [8–10, 14]. The SMG is composed primarily of ductal and acinar cells that can easily be distinguished by morphology and/or expression of cell-specific markers (Fig 1). Following 7 days of SMG duct ligation, expression of the acinar cell marker aquaporin-5 (AQP5), an apical membrane water channel, is dramatically reduced as acinar cells atrophy (Fig 1A vs. 1B) [17, 50]. Additionally, SMG duct ligation leads to enhanced immune cell infiltration, as judged by expression of the pan-immune cell marker CD45 (Fig 1E) [8, 10], and the loss of ductal secretory granules (Fig 1H). Once the SMG excretory duct is deligated the gland begins to regenerate and, after a 28 day recovery period, expression of the acinar cell marker AQP5 returns to levels (Fig 1C) comparable to control glands (Fig 1A). Additionally, the number of immune cells returns to levels (Fig 1F) comparable to control glands (Fig 1D). Ductal secretory granules also return after deligation (Fig 1I) and duct morphology resembles unligated control glands (Fig 1G).

### Reversible upregulation of TGF-β isoforms and TGF-β R1 occurs in the SMG following excretory duct ligation and deligation

**Fig 2:**
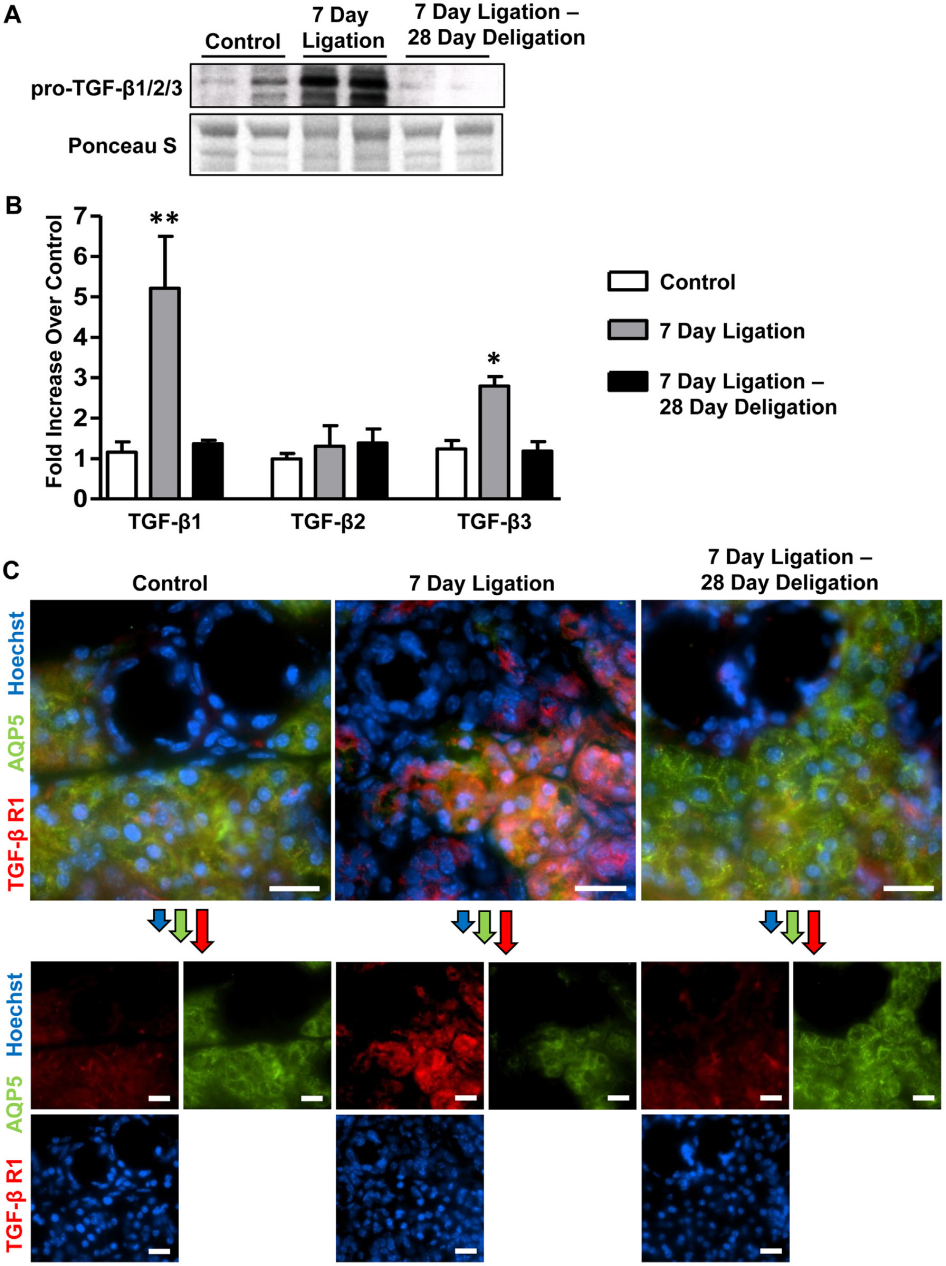
Upregulation of TGF-β1, TGF-β3 and TGF-β R1 in response to SMG duct ligation is reversible upon deligation. (A) Western analysis of whole gland lysates shows upregulation of pro-TGF-β1/2/3 expression (top) in response to 7 day SMG duct ligation that is reversible following deligation and recovery for 28 days. Ponceau S staining (bottom) shows equal amounts of total protein in each well. (B) RT-PCR analysis of cDNA prepared from whole SMGs shows significant upregulation of TGF-β1 and TGF-β3, but not TGF-β2, mRNA expression after 7 days of SMG excretory duct ligation (grey bars), as compared to contralateral control glands (white bars). When ducts were ligated for 7 days then deligated for 28 days (black bars), expression levels of TGF-β1 and TGF-β3 mRNA return to control levels, whereas TGF-β2 mRNA levels remain unchanged. Data represent means ± S.E.M. (n = 7 for control and 7 day ligation, n = 5 for 7 day ligation, deligation and a 28 day recovery), where *P<0.05 and **P<0.01 indicate significant differences in mRNA expression, as compared to control. (C) Dual-immunofluorescence analysis of 8 μm frozen SMG sections for control, 7 day ligation and 7 day ligation followed by deligation and recovery for 28 days reveals that TGF-β R1 (red) expression is upregulated primarily in acinar cells (marked by residual AQP5 expression; green) after a 7 day SMG duct ligation, whereas little staining is visible in ductal cells. After a 7 day duct ligation followed by deligation and a 28 day recovery, TGF-β R1 expression levels are similar to control. Hoechst nuclear stain in blue and scale bar = 20 μm. Images are representative of results from at least 3 independent experiments.

TGF-β signaling has been shown to regulate tissue fibrosis and regeneration in a number of tissues including liver, lung and kidney [22–25]. However, a role for TGF-β signaling in response to SMG duct ligation and deligation has not been defined. Western analysis using an antibody that recognizes all three TGF-β isoforms shows substantial TGF-β upregulation in response to a 7 day duct ligation. After duct deligation and a 28 day recovery, the levels of pro-TGF-β1/2/3 return to levels comparable to control glands (Fig 2A). RT-PCR analysis of cDNA prepared from whole SMGs shows that expression of mRNA to TGF-β1 and TGF-β3, but not TGF-β2, is significantly increased in the SMG following 7 days of duct ligation, as compared to control glands (Fig 2B). Furthermore, TGF-β1 and TGF-β3 upregulation in response to duct ligation was fully reversible upon duct deligation and a 28 day recovery period, where expression levels of these cytokines returned to control levels (Fig 2B). In addition to TGF-β1 and TGF-β3, TGF-β R1 expression was significantly increased following SMG duct ligation, as compared to control glands, and returned to control levels 28 days after deligation (Fig 2C). Dual-immunofluorescence staining of TGF-β R1 and the acinar marker AQP5 revealed that the expression of TGF-β R1 in the ligated SMG was restricted to acinar cells, similar to control glands.

### Reversible upregulation of canonical and non-canonical TGF-β signaling pathways in response to excretory duct ligation and deligation

**Fig 3:**
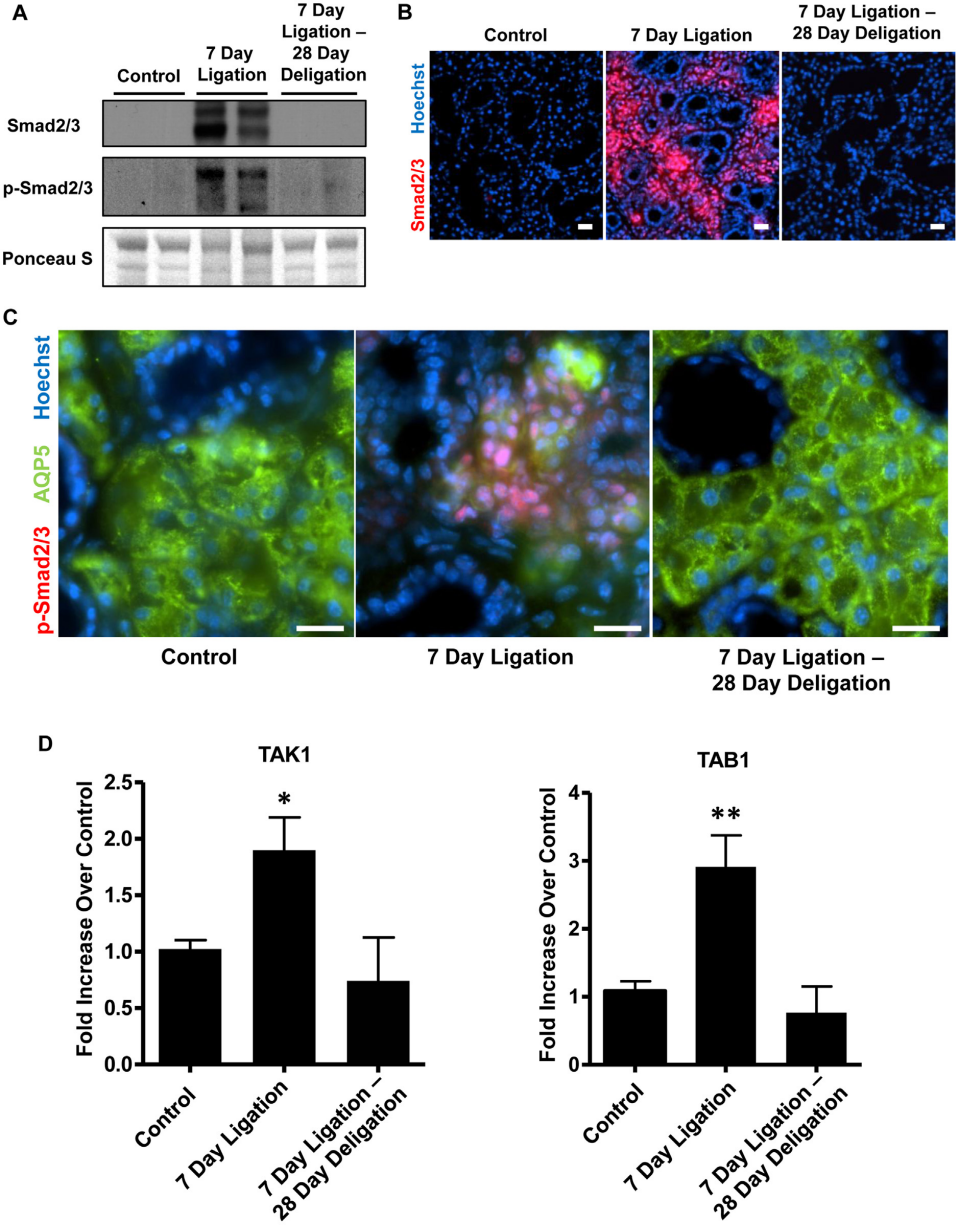
Upregulation of Smad2/3, TAK1 and TAB1 in response to SMG duct ligation is reversible upon duct deligation. (A) Western blot analysis of whole gland lysates from unligated control SMG, SMG after a 7 day duct ligation or a 7 day ligation followed by deligation and a 28 day recovery. Duct ligation increases Smad2/3 expression (top) and phospho-Smad2/3 levels (middle) that return to control levels after deligation and recovery. Ponceau S staining (bottom) shows equal amounts of total protein in each well. Immunofluorescence analysis revealed that increases in (B) Smad2/3 (red) expression and (C) p-Smad2/3 (red) levels after a 7 day duct ligation are restricted to acinar cells (marked by residual AQP5 expression; green), where p-Smad2/3 is localized to the nucleus, as determined by colocalization with Hoechst nuclear stain (blue). Smad2/3 and p-Smad2/3 returned to control levels after deligation of SMG ducts and recovery. Images are representative of results from at least 3 independent experiments and scale bar = 20 μm. (D) RT-PCR analysis of whole gland lysates shows increased TAK1 and TAB1 mRNA expression following a 7 day duct ligation, which was reversed to control levels following deligation and a 28 day recovery. Data represent means ± S.E.M. (n = 6 for control, n = 8 for 7 day ligation, n = 5 for 7 day ligation, deligation and a 28 day recovery), where *P<0.05 and **P<0.01 indicate significant differences in mRNA expression, as compared to control SMG.

Canonical TGF-β signaling occurs following the binding of TGF-β to its cognate receptor to induce the phosphorylation and activation of the intracellular transcription factors Smad2 and Smad3, which then translocate to the nucleus to activate downstream targets of the TGF-β signaling cascade [30, 51]. Utilizing an antibody that recognizes both Smad2 and Smad3, Western analysis indicates that the expression of Smad2/3 in whole gland lysates from control SMGs is highly upregulated following 7 days of duct ligation (Fig 3A). The level of Smad2/3 activation, as measured by the amount of phosphorylated-Smad2/3 (p-Smad2/3) in whole gland lysates, was also highly increased following SMG duct ligation (Fig 3A). After duct deligation and a 28 day recovery, the levels of Smad2/3 and p-Smad2/3 return to levels comparable to control glands (Fig 3A). Immunofluorescence analysis revealed that the increased expression of Smad2/3 (Fig 3B) and p-Smad2/3 (Fig 3C) induced by SMG duct ligation is localized to acinar cells, a response that was reversed upon deligation and a 28 day recovery (Fig 3B and 3C), similar to TGF-β R1 expression patterns (Fig 2B). Dual-immunofluorescence staining of p-Smad2/3 and AQP5 revealed that the expression of p-Smad2/3 following a 7 day duct ligation colocalized with Hoechst nuclear stain in acinar cells (Fig 3C).

In addition to canonical signaling through the activation of Smad transcription factors, non-canonical TGF-β signaling occurs through the activation of the mitogen-activated protein kinase-3 (MAP3K) TAK1 and the TAK1-binding protein TAB1 [52]. RT-PCR analysis reveals significant upregulation of both TAK1 and TAB1 mRNA expression following 7 days of SMG duct ligation and expression returned to levels comparable to unligated control glands following deligation and a 28 day recovery (Fig 3D).

### Reversible upregulation of Snail and Slug in response to duct ligation and deligation

**Fig 4:**
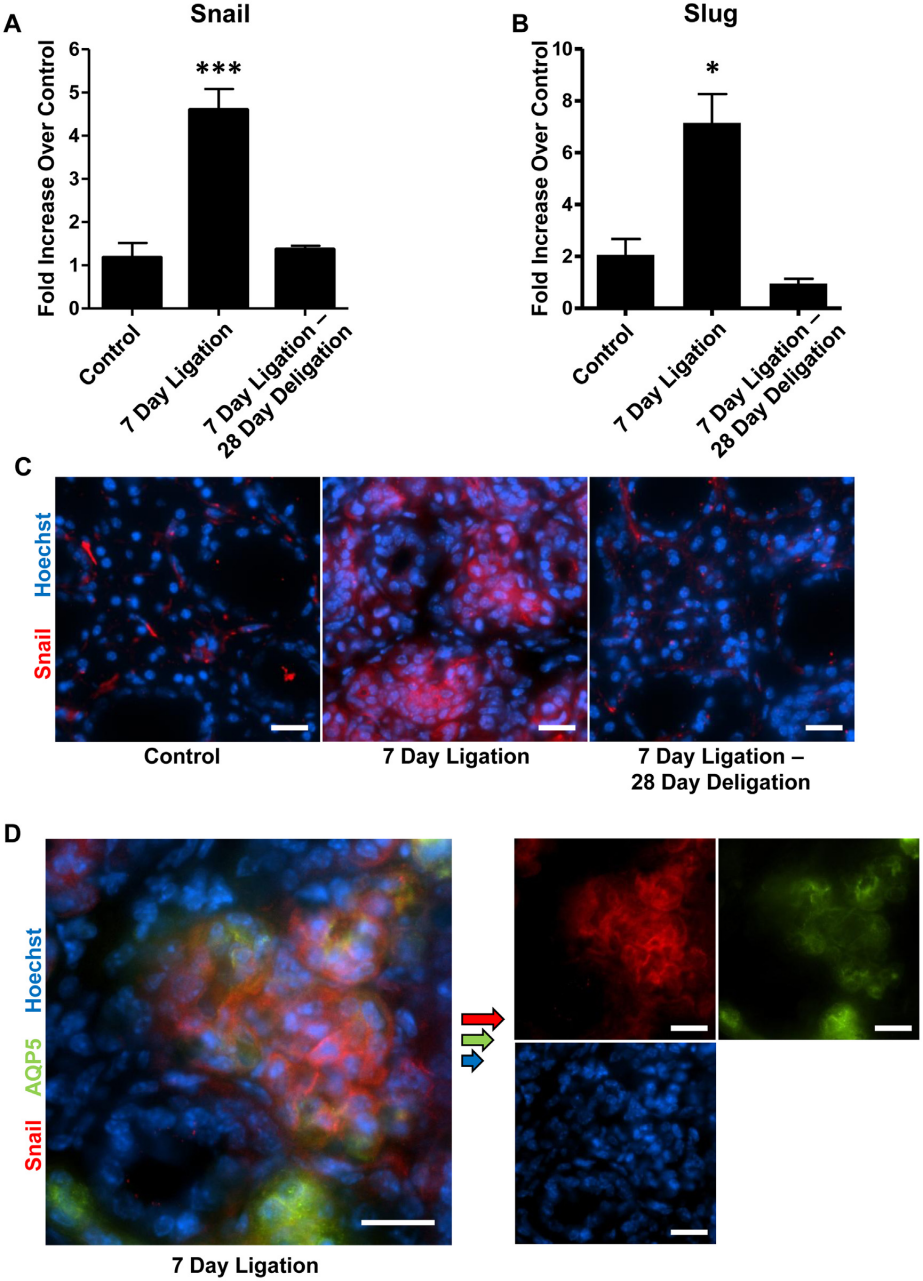
Upregulation of Snail and Slug in response to SMG duct ligation is reversible upon duct deligation. RT-PCR analysis of cDNA prepared from whole SMGs shows significant upregulation of (A) Snail and (B) Slug mRNA after a 7 day duct ligation, which is reversed to control levels after deligation and a 28 day recovery. Data represent means ± S.E.M. (n = 4 for control, n = 8 for 7 day ligation, n = 5 for 7 day ligation, deligation and a 28 day recovery), where ***P<0.001 and *P<0.05 indicate significant differences in mRNA expression, as compared to control SMG. (C) Immunofluorescence analysis reveals that Snail (red) expression is primarily upregulated in acinar cells after a 7 day duct ligation, and returns to control levels after deligation and recovery. (D) Dual-immunofluorescence staining confirmed the colocalization of Snail (red) with the acinar marker AQP5 (green) following a 7 day duct ligation. Hoechst nuclear stain in blue and scale bar = 20 μm. Images are representative of results from at least 3 independent experiments.

Smad2/3 phosphorylation and translocation to the nucleus mediates TGF-β-induced cellular responses through transcriptional activation of other DNA-binding transcription factors, including Snail (Snai1) and Slug (Snai2) [53]. The transcription repressor Snail is a primary target for regulation through direct binding of Smad2/3 to the *Snail* promoter [54, 55]. Following 7 days of SMG duct ligation, both Snail and Slug mRNA expression in whole SMGs was significantly increased, as compared to control glands, and expression was reversed to control levels after ductal deligation and a 28 day recovery (Fig 4A and 4B). Immunofluorescence analysis of Snail localization following SMG duct ligation and deligation revealed a similar pattern to Smad2/3 and TGF-β R1, with Snail upregulation primarily restricted to acinar cells (Fig 4C). Dual-immunofluorescence analysis confirmed the colocalization of Snail and AQP5 following a 7 day duct ligation (Fig 4D).

### Reversible upregulation of E-cadherin in response to SMG duct ligation and deligation

**Fig 5:**
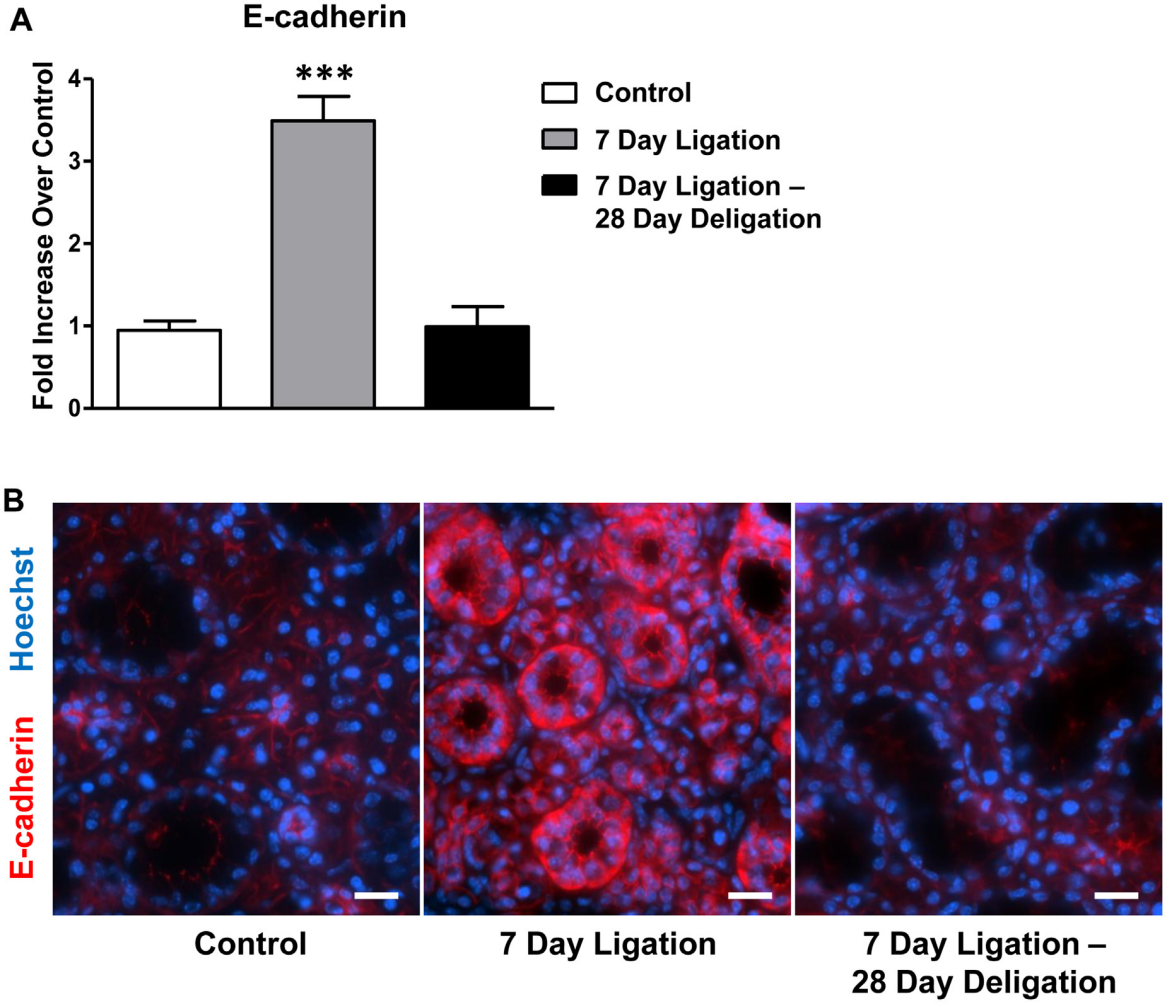
E-cadherin upregulation in response to SMG duct ligation is reversible after duct deligation. (A) RT-PCR analysis of cDNA prepared from whole SMGs shows significant upregulation of E-cadherin mRNA after a 7 day duct ligation (grey bar), which is reversed to control levels (white bar) after deligation and a 28 day recovery (black bar). Data represent means ± S.E.M. (n = 8 for control, n = 7 for 7 day ligation, n = 5 for 7 day ligation, deligation and a 28 day recovery), where ***P<0.001 indicates a significant difference in mRNA expression, as compared to control SMG. (B) Immunofluorescence analysis reveals that E-cadherin (red) was significantly upregulated in SMG ducts after a 7 day ligation, and returned to control levels after deligation and recovery. Hoechst nuclear stain in blue and scale bar = 20 μm. Images are representative of results from at least 3 independent experiments.

Previous reports have described a role for TGF-β in EMT and Snail has been shown to be a primary effector in this pathway by repressing E-cadherin expression [32, 41]. Because our data show that upregulation of the transcriptional repressor Snail occurs after SMG duct ligation (Fig 4) and E-cadherin expression is suppressed by Snail during EMT [32], we investigated whether E-cadherin expression is altered in response to SMG ligation and deligation. Interestingly, following 7 days of duct ligation, E-cadherin mRNA expression was significantly upregulated (Fig 5A), despite the finding that TGF-β signaling molecules and Snail expression were increased following SMG duct ligation (Figs 2–4). Immunofluorescence analysis of E-cadherin localization after SMG duct ligation revealed that E-cadherin was highly upregulated in ductal cells, suggesting that ductal cells maintain epithelial integrity (Fig 5B). It seems likely that upregulation of E-cadherin in ductal cells (Fig 5B) is responsible for the overall upregulation of E-cadherin in whole gland cell lysates (Fig 5A). Following deligation and a 28 day recovery, the expression of E-cadherin returned to levels comparable to control glands (Fig 5A and 5B).

### Glandular fibrosis resulting from SMG duct ligation is resolved following ductal deligation

**Fig 6:**
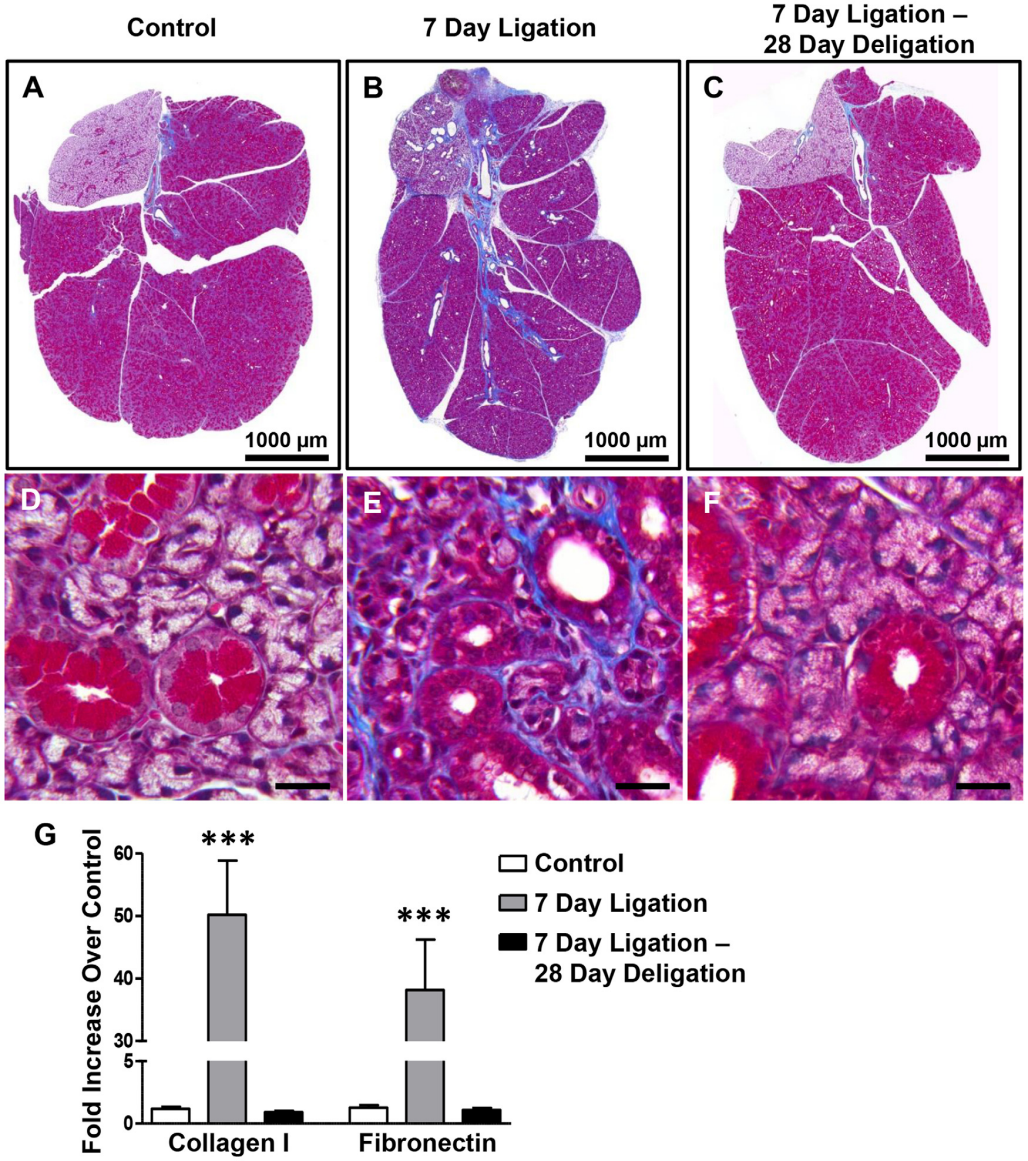
Glandular fibrosis following SMG duct ligation is resolved following deligation. SMGs from unligated controls (A, D), after a 7 day duct ligation (B, E) or a 7 day ligation followed by deligation and a 28 day recovery (C, F) were subjected to Masson’s trichrome staining to analyze collagen deposition (blue). A 7 day duct ligation resulted in blue staining of collagen fibers around blood vessels and large saliva collecting ducts (B), as compared to controls (A). At 400X magnification, heavy deposition of collagen fibers can be seen in the interstitial area around acinar and ductal cells after a 7 day duct ligation (E), as compared to controls (D). Following ductal deligation and a 28 day recovery (C, F), collagen deposition returns to levels similar to control SMGs (A, D). Images are representative of results from at least 3 independent experiments. (A-C) scale bar = 1,000 μm, (D-F) scale bar = 20 μm. (G) RT-PCR analysis of cDNA prepared from whole SMGs shows extensive upregulation of collagen 1 and fibronectin mRNAs after a 7 day duct ligation (grey bars), which is reversed to control levels (white bars) after deligation and a 28 day recovery (black bars). Data represent means ± S.E.M. (n = 8 for control, n = 7 for 7 day ligation, n = 5 for 7 day ligation, deligation and a 28 day recovery), where ***P<0.001 indicates a significant difference in mRNA expression, as compared to control SMGs.

Previous studies have demonstrated that SMG duct ligation can increase collagen deposition and glandular fibrosis [20]. Additionally, conditional overexpression of TGF-β1 in the salivary glands leads to glandular fibrosis [56]. To investigate fibrosis progression and resolution in the murine duct ligation/deligation model, SMG ducts were ligated for 7 days followed by deligation and a 28 day recovery. After 7 days of duct ligation, collagen deposition assayed by Masson’s Trichrome staining (blue) was substantially increased around blood vessels and large collecting ducts of SMG as well as within the interlobular stroma (Fig 6B), as compared to control SMG (Fig 6A). Analysis at higher magnification revealed that duct ligation also caused extensive collagen deposition in the interstitial areas around acinar cells and ductal cells (Fig 6E), as compared to control SMG (Fig 6D), suggesting that active extracellular matrix (ECM) production and deposition occurred in these regions of the SMG. Following ductal deligation and a 28 day recovery (Fig 6C and 6F), glandular fibrosis was resolved and collagen deposition around blood vessels, collecting ducts and interstitial areas resembled control glands (Fig 6A and 6D). RT-PCR analysis of cDNA prepared from whole SMGs revealed significant upregulation of collagen 1 and fibronectin mRNA after a 7 day duct ligation that returned to control levels after deligation and a 28 day recovery (Fig 6G).

### Treatment with TGF-β R1 inhibitors attenuates upregulation of fibrosis markers in response to SMG duct ligation

**Fig 7:**
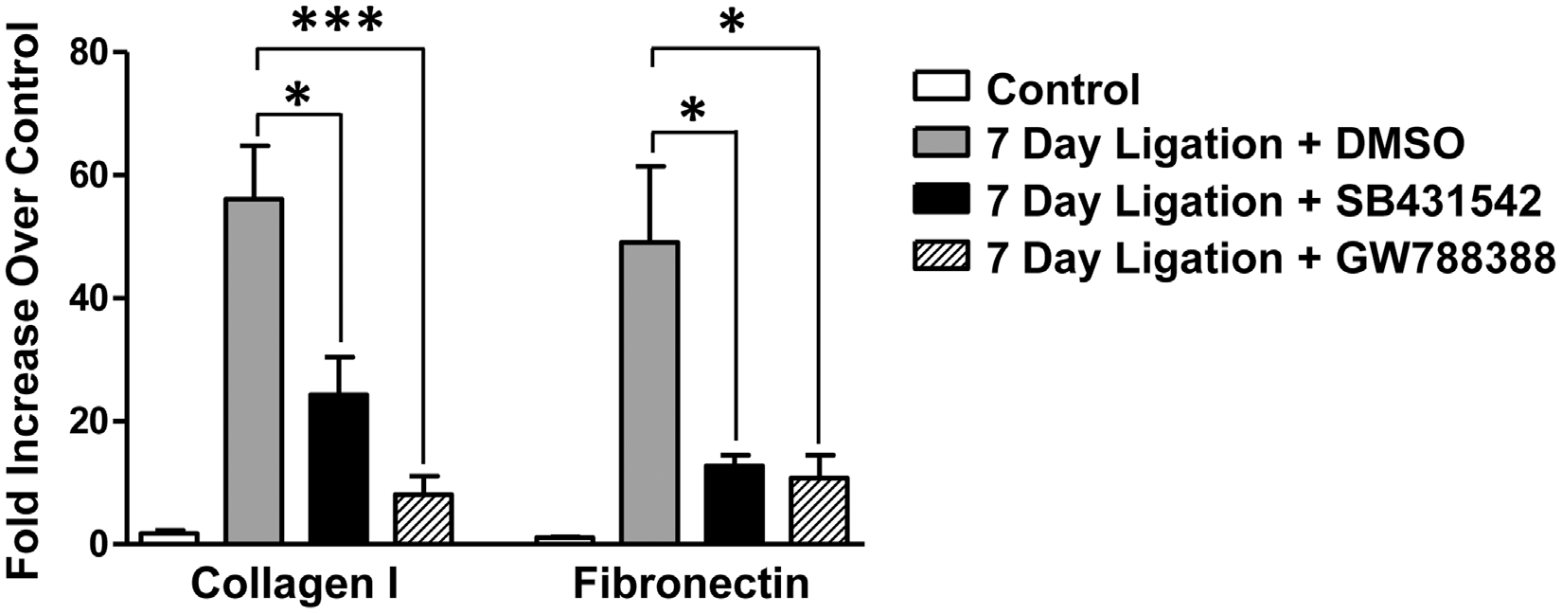
TGF-β R1 inhibitors SB431542 and GW788388 attenuate duct ligation-induced upregulation of fibrosis markers. RT-PCR analysis of 7 day ligated and contralateral unligated control SMGs shows significant attenuation of 7 day duct ligation-induced collagen 1 and fibronectin mRNA upregulation in mice treated with either SB431542 (20 mg/kg mouse weight) or GW788388 (2 mg/kg mouse weight), as compared to DMSO-treated controls. Data represent means ± S.E.M. (n = 6 for DMSO, n = 6 for SB431542, n = 5 for GW788388), where *P<0.05 and ***P<0.001 indicate significant differences in mRNA expression, as compared to DMSO-treated controls.

The small molecule TGF-β R1 inhibitors SB431542 and GW788388 have been shown to reduce TGF-β-mediated signaling and tissue fibrosis in mouse models of kidney, liver and lung fibrosis [57–60]. To determine whether TGF-β R1 inhibition reduces 7 day duct ligation-induced SMG fibrosis, mice were treated with SB431542 (20 mg/kg mouse weight), GW788388 (2 mg/kg mouse weight) or DMSO directly after SMG duct ligation and on day 4 post-ligation. RT-PCR analysis of 7 day ligated and contralateral unligated glands revealed significant attenuation of duct ligation-induced collagen 1 and fibronectin mRNA upregulation following treatment with either SB431542 or GW788388, as compared to DMSO-treated ligated glands (Fig 7).

## Discussion

TGF-β participates in diverse biological processes including development, inflammation, fibrosis, tissue regeneration and EMT [23, 25, 41, 61, 62]. In salivary glands, previous studies have shown that TGF-β isoforms can modulate branching morphogenesis during salivary gland development [63]. Additionally, *in vitro* studies have shown that TGF-β signaling modulates the formation of acinar units by primary murine SMG cells and the human salivary gland (HSG) cell line [64, 65], suggesting that TGF-β plays a role in regeneration of salivary acini *in vivo*. Overexpression of TGF-β1 in murine salivary glands has been shown to significantly disrupt salivary gland development and function, likely due to glandular atrophy and fibrosis [56]. Conversely, inhibition of TGF-β signaling in the salivary gland through conditional knockout of TGF-β R1 also leads to salivary dysfunction through the development of multifocal lymphocytic inflammation of the salivary glands [66]. Global knockout of TGF-β1 in mice leads to a severe inflammatory phenotype characterized by lymphocytic infiltration of the heart, lung and salivary glands and premature death within 4 weeks of age [67]. Further investigation has revealed that TGF-β1^-/-^ mice develop nuclear autoantibodies, glandular atrophy, loss of acinar cells and significant inflammatory lesions in the salivary gland, all of which are traits similar to the human autoimmune disorder Sjögren’s syndrome [68, 69]. While attempts to determine TGF-β levels in salivary glands of SS patients have yielded conflicting data [70, 71], studies have shown that TGF-β expression increases in patients suffering from chronic obstructive sialadenitis, a human condition caused by obstruction of the SMG excretory duct [72, 73]. Because the rodent model of SMG duct ligation closely mimics obstructive sialadenitis [74], the findings presented in this paper have clinical relevance.

Tissue fibrosis is typically associated with chronic inflammation where molecular signals from unrepaired injured tissue stimulate resident fibroblasts, epithelial cells (through EMT) or bone marrow-derived fibrocytes to differentiate into myofibroblasts [75, 76]. Overproduction and deposition of ECM components, such as collagen 1 and fibronectin, progressively replace normal parenchyma and disrupt tissue morphology and function [75]. *In vitro* studies have shown that TGF-β stimulates the production of collagen and fibronectin, key proteins in the development of fibrosis [77, 78]. *In vivo*, TGF-β also has been shown to stimulate fibrogenesis in animal models of liver, lung and kidney fibrosis [79–81]. Salivary gland fibrosis following radiation exposure during treatment of head and neck cancers contributes to long term hyposalivation in patients [82, 83] and increased TGF-β expression has been reported in patients suffering from radiation-induced xerostomia [84]. Additionally, TGF-β has been shown to initiate fibrosis following radiation treatment of the skin [85], suggesting that TGF-β may contribute to radiation-induced salivary gland fibrosis. The results from this study show that significant collagen deposition and upregulation of collagen 1 and fibronectin mRNA (Figs 6–7) in the SMG following duct ligation occurs concurrently with increased expression of TGF-β signaling components (Figs 2–4). Moreover, ligation-induced SMG fibrosis is attenuated following administration of the TGF-β R1 inhibitors SB431542 and GW788388 (Fig 7). These small molecule TGF-β R1 inhibitors have been shown to inhibit TGF-β signaling through competitive antagonism of TGF-β R1 kinase activity, thereby preventing phosphorylation and activation of Smad transcription factors [86, 87] suggesting that this canonical TGF-β signaling pathway plays a major role in duct-ligation induced fibrosis. The remarkable ability of the duct-ligated SMG to regenerate following deligation makes this an ideal model to investigate the molecular pathways involved in fibrosis resolution. Identifying mechanisms that modulate the expression or function of proteins which aid in fibrosis resolution (*i*.*e*., matrix metalloproteases (MMPs), tissue inhibitors of metalloproteases (TIMPs) and hepatocyte growth factor (HGF) [88–90]) in the murine duct-ligated SMG could help develop strategies to reduce salivary gland fibrosis in humans that occurs in chronic obstructive sialadenitis or following radiation treatment for head and neck cancer.

In addition to its role in stimulating myofibroblasts during fibrosis, TGF-β has significant effects on epithelial cells. TGF-β signaling in epithelial cells has been shown to induce migration, reduce proliferation and stimulate apoptosis [91–93]. Interestingly, the data presented in this paper suggest that TGF-β signaling is very active in salivary acinar cells during duct ligation (Figs 2–4). Previous studies have demonstrated the presence of TGF-β in saliva where it is hypothesized to function in maintaining immune homeostasis in the oral cavity and esophagus [94, 95]. Furthermore, immunohistochemical studies have shown that TGF-β is localized to ductal cells in the salivary glands of humans and mice under normal and pathological circumstances [65, 70, 71]. Our data suggest that TGF-β R1 is primarily expressed in salivary acinar cells (Fig 2C) and these cells also demonstrate upregulation of molecules in the TGF-β signaling cascade following ductal ligation (Figs 3 and 4). Perhaps ductal cells secrete TGF-β during normal salivary gland function which then accumulates in the lumen after the gland is ligated, whereupon elevated TGF-β levels activate the upregulated TGF-β R1 on salivary acinar cells. Previous studies have shown that some subsets of ductal cells proliferate in response to SMG duct ligation while acinar cells downregulate expression of AQP5 and eventually undergo apoptosis [11, 15, 19]. Thus, the altered expression of TGF-β R1 in salivary acinar cells may be a contributing factor to the known responses of salivary epithelium to SMG duct ligation.

The ability of TGF-β to induce the transition of epithelial cells into a mesenchymal phenotype has been extensively studied both *in vitro* [41, 65, 96–99] and *in vivo* [100–104], and is widely regarded as a necessary process in embryogenesis [105]. However, the *in vivo* significance of post-embryogenesis EMT has been debated [106–108]. In exocrine glands, EMT has been shown to play a role in the repair of lacrimal glands following IL-1-induced injury [109] and, in the pancreas, EMT induces the formation of insulin-secreting beta cells from pancreatic acinar cells [110, 111]. Other studies have provided *in vivo* evidence of TGF-β-induced EMT in the kidney, liver and lungs [100, 103, 104]. During EMT, epithelial cells downregulate the expression of proteins necessary for epithelial function, such as the cell adhesion molecule E-cadherin and the tight junction proteins ZO-1 and claudin, and upregulate proteins that contribute to mesenchymal function, such as the intermediate fiber vimentin and α-smooth muscle actin [37, 41]. TGF-β induces EMT through the phosphorylation and activation of Smad family transcription factors that then induce the upregulation of other transcriptional regulators, such as the zinc-finger transcription repressors Snail and Slug [32, 33, 54]. Snail is a well-described repressor of E-cadherin expression [32, 42, 43] and overexpression of Snail induces EMT in several types of epithelial cells [32]. TGF-β-induced TAK1 activation, which is thought to occur through TAK1/TAB1 interaction with TNF receptor-associated factor 6 (TRAF6) and downstream NF-κB activation, has been shown to regulate EMT in both human lung epithelial and peritoneal-derived mesothelial cells [35, 36, 112]. Our results show increased Smad2/3 and TAK1/TAB1 expression (Fig 3) as well as upregulation of Snail and Slug expression following ductal ligation (Fig 4), which suggests that TGF-β signaling is increased in salivary epithelial cells during injury. Of particular interest is the differential expression pattern of Snail in response to duct ligation where ductal cells appear to have low expression levels of Snail, whereas acinar cells have much higher expression levels (Fig 4C and 4D). This acinar localization of Snail is in agreement with Smad2/3 localization (Fig 3B and 3C), both of which are likely the result of the upregulation and localization of TGF-β R1 in acinar cells caused by SMG duct ligation (Fig 2C). If TGF-β-induced EMT occurs in duct-ligated SMG, one would expect to find E-cadherin downregulation in the gland [32]. However, our results show significant upregulation of E-cadherin following a 7 day SMG duct ligation (Fig 5A). It seems likely that upregulation of E-cadherin in ductal and not acinar cells (Fig 5B) is responsible for the overall upregulation of E-cadherin in whole gland cell lysates (Fig 5A). Interestingly, previous studies have shown that E-cadherin expression increases in kidney tubules following ureteral ligation [113]. Taken together, these data suggest that during SMG duct ligation, salivary epithelium (acinar and ductal cells) have diverse responses which could be driven in part by differential capacities for TGF-β signaling. Whereas salivary acinar cells upon SMG duct ligation exhibit decreased expression of AQP5 (Fig 1) [17] and cellular atrophy (Fig 1) [50] and increased expression of TGF-β signaling molecules (Figs 2–4), ductal cells exhibit increased E-cadherin expression (Fig 5) and proliferation [18, 19]. Perhaps the increase in E-cadherin expression in ductal cells is a protective response. Previous studies have demonstrated a role for E-cadherin in cell survival, where conditional knockout of E-cadherin in alveolar epithelial cells in the mammary gland led to significant apoptosis and glandular dysfunction [114, 115]. Whether salivary acinar cells actually undergo EMT during duct ligation remains to be determined, however, it seems likely that TGF-β signaling contributes to the observed changes in acinar cell morphology that occur upon duct ligation.

The results from this study provide evidence of a role for TGF-β during mature salivary gland damage and regeneration. We show significant upregulation of TGF-β1 and TGF-β3 as well as TGF-β R1 in response to SMG duct ligation (Fig 2). Furthermore, we provide evidence for active TGF-β signaling in salivary acinar epithelial cells caused by duct ligation, as indicated by increases in the expression and phosphorylation of Smad2/3 and the expression of its downstream target Snail (Figs 3 and 4). This study also provides evidence that SMG duct ligation causes fibrosis (Fig 6), concurrent with enhanced TGF-β signaling in acinar cells (Figs 2–4) that can be attenuated following administration of small molecule TGF-β R1 inhibitors (Fig 7). We also describe the reversibility of these cellular responses upon ductal deligation and recovery, thereby demonstrating the capacity of the SMG to regenerate following damage and fibrosis. Although it has classically been used to study inflammatory and regenerative pathways, SMG duct ligation-induced damage appears to be an ideal model for investigating mechanisms that initiate and resolve salivary gland fibrosis. The observed differential responses of salivary acinar and ductal cells to TGF-β also indicate that this model should be useful for *in vivo* studies of TGF-β signaling in epithelial cells. Further insights into TGF-β signaling in duct-ligated SMG could identify novel therapeutic targets for salivary gland fibrosis associated with obstructive sialoadenitis and radiation-induced damage to the salivary gland in humans.
